# Editorial: The role of negative immune checkpoints in the treatment of systemic lupus erythematosus and rheumatoid arthritis

**DOI:** 10.3389/fimmu.2025.1582125

**Published:** 2025-03-11

**Authors:** Jitian Li, Jun Liu, Liping Dai, Xinzhi Wang, Yun Shi

**Affiliations:** ^1^ Clinical Medical Center of Tissue Engineering and Regeneration, The Third Affiliated Hospital of Xinxiang Medical University, Xinxiang Medical University, Xinxiang, China; ^2^ Laboratory of Molecular Biology, Luoyang Orthopedic Hospital of Henan Province (Orthopedic Hospital of Henan Province), Zhengzhou, China; ^3^ State Key Laboratory of Natural Medicines, New Drug Screening and Pharmacodynamics Evaluation Center, China Pharmaceutical University, Nanjing, China; ^4^ Henan Institute of Medical and Pharmaceutical Sciences & Henan Key Medical Laboratory of Tumor Molecular Biomarkers, Zhengzhou University, Zhengzhou, China; ^5^ Institute for Biomedicine and Glycomics, Griffith University, Gold Coast, Australia

**Keywords:** autoimmune diseases, immune checkpoint, systemic lupus erythematosus, rheumatoid arthritis, signaling pathway, risk factor

Autoimmune diseases arise from a complex interplay of genetic, environmental, and immunological factors, leading to dysregulated immune responses and chronic inflammation. Despite significant advances in understanding the pathogenesis of autoimmune diseases, many questions remain regarding their etiology, progression, and therapeutic targets. This Research Topic comprises four articles exploring potential negative immune checkpoints and therapeutic targets for autoimmune diseases such as systemic lupus erythematosus (SLE) and rheumatoid arthritis (RA), offering valuable insights into potential biomarkers, therapeutic strategies, and disease management approaches.

SLE and RA are both chronic inflammatory diseases caused by abnormal immune system. Tang et al. review the role of hypoxia-inducible factor-1α (HIF-1α) in inflammatory autoimmune diseases including SLE and RA. As a primary metabolic sensor widely expressed in both immune and non-immune cells, HIF-1α promotes the proliferation and differentiation of adaptive immune cells and the secretion of inflammatory cytokines. Its expression is elevated in autoimmune diseases such as SLE and RA, implicating it in disease pathology. This review highlights the potential of HIF-1α as a therapeutic target and biomarker in autoimmune diseases.

Hossen et al. examine the role of cytotoxic T-lymphocyte antigen 4 (CTLA-4) in autoimmune diseases. CTLA-4, a key immune checkpoint molecule, maintains immune homeostasis by modulating effector T-cell activity and enhancing regulatory T-cell function. This review discusses the structure, expression, and molecular mechanisms of CTLA-4, emphasizing its potential as a therapeutic target in various autoimmune diseases. It highlights the ritical role of CTLA-4 in immune regulation and its potential application in the treatment of autoimmune diseases.

In addition to immune checkpoints, immune signaling pathways also play critical roles in autoimmune diseases. Liu and Pu review the role of the cyclic GMP-AMP synthase-stimulator of the interferon gene (cGAS-STING) signaling pathway in autoimmune and autoinflammatory diseases. cGAS-STING detects cytosolic DNA and triggers inflammatory responses via type I interferons (IFN-I) and other inflammatory factors. While crucial for host defense, dysregulated cGAS-STING signaling contributes to excessive inflammation and autoimmunity. Recent research has linked aberrant activation of this pathway to autoimmune diseases, including SLE and RA. This review provides a comprehensive overview of cGAS-STING activation mechanisms, its pathological implications in autoimmune diseases, and emerging therapeutic strategies, including small molecule inhibitors, targeting this pathway.

Identifying potential risk factors of autoimmune diseases is another key area of investigation. Osteonecrosis of the femoral head (ONFH) is a severe complication of SLE. By analyzing clinical records of 793 female SLE patients with a predictive nomogram model, Xu et al. identify ten independent risk factors for ONFH. While many of these ten risk factors are related to abnormal activation of immune system and inflammatory response, menstrual abnormalities emerge as a previously unrecognized risk factor. The proposed nomogram demonstrates strong predictive performance, with an area under the ROC curve of 0.826. Subgroup analysis further highlights the increased ONFH risk in younger patients and those receiving high-dose glucocorticoids. This study provides a clinically applicable tool for risk assessment and underscores the importance of monitoring reproductive health in female SLE patients.

In summary, these four articles offer critical insights into autoimmune disease mechanisms and clinical applications. Mechanistic studies on cGAS-STING, CTLA-4, and HIF-1α elucidate key pathways that contribute to immune dysregulation and inflammation, while the identification of novel risk factors, such as menstrual abnormalities in ONFH, enhances risk assessment in autoimmune disease management. By integrating molecular immunology with epidemiological findings, this Research Topic advances our understanding of autoimmune diseases such as SLE and RA and paves the way for improved patient care and therapeutic development.

